# Inversion in Suspended Animation during Anæsthesia

**Published:** 1888-04

**Authors:** 


### INVERSION IN SUSPENDED ANIMATION DURING ANÆSTHESIA.

Professor JULIAN J. CHISOLM, in a paper entitled "A Very Valuable Lesson for Those who Use Anæsthetics," read before the Baltimore Academy of Medicine on December 6, 1887, relates several cases in which inversion of the patient was successfully used in suspended animation from chloroform anæsthesia.

The first and most interesting case was that of a boy three years of age, upon whom Dr. Chisolm wished to operate for cancer of the left eye. When the anæsthesia was complete the operation was begun, but before much progress had been made respiration suddenly ceased. The face assumed a death-like pallor, and the pulse disappeared from the wrist. Immediately the child was suspended by the feet, with body and head hanging down at an inclination of 70 degrees, while an assistant made chest-compression for artificial respiration. After a few minutes signs of feeble respiratory movement were noticed, a slight throbbing of the neck-vessels was detected, and in time the child evinced its return to consciousness by crying. The child would not permit the eye to be touched without moving the head, and as the operation had to be completed chloroform was again administered. Before the operation was begun a second time the child again stopped breathing and the pulse disappeared. The body, apparently of a dead child, was once more hung up by the feet, so as to allow blood to gravitate toward the anæmic head and brain, but with no further attempts at artificial respiration. After a few minutes the large vessels at the root of the neck showed some fullness; then a slight thrill, and after this the first attempt at a thoracic movement appeared. In ten minutes breathing was sufficiently strong to allow the child to cry again.

Chloroform was given a third time, and was very soon followed by suspended animation, and death now seemed to be complete. The child was again hung up by the feet. After a little more than five minutes a faint effort at respiration was seen, and when it seemed fully established, though insensibility continued, the child was replaced on the table. As soon as the body was placed in the horizontal position the pulse, not yet strong, disappeared from the wrist, and respiration ceased, requiring a renewal of the suspension. This was repeated three or four times.

Finally the eye was enucleated while the child was suspended with the head downward. It was fully a quarter of an hour after the operation was completed, and the eye bandaged, before the child could be trusted in the recumbent posture; and in all the child was suspended about three-quarters of an hour.

For ten years Dr. Chisolm has not given ether to any patient. For painful operations of very short duration he uses bromide of ethyl, but for all other he gives chloroform exclusively. He has administered chloroform about 10,000 times, and has never had a death. In the administration of the anæsthetic certain rules are followed: All clothing must be loose around the neck. With adults an ounce of whisky is given in advance, though this cardiac stimulant is omitted in persons under thirty years of age, unless the patient be feeble. In old persons, with feeble and irregular heart, the amount of whisky is increased. The whisky is always put into the patient's stomach, where it can be used if needed, and where it can do no harm if it is not needed. Dr. Chisolm has never had occasion to inject whisky or ether into the rectum or under the skin.

Chloroform is administered with the patient lying on his back, and, as soon as narcosis is induced, the pillow is taken from under his head, so as to have him lie in an absolutely horizontal position. Should snoring occur, indicating some difficulty in the pharyngeal breathing, the chin is drawn forcibly upward. This elevation pulls the anterior wall of the pharynx, with the hyoid bone and root of tongue, forward, making for the air a clear and straight passage from the nose into the lungs. By this movement of the chin respiration becomes immediately quiet and easy. The pulling up of the chin is a much more efficient and convenient means of pulling the root of the tongue forward than by pulling out the tongue with a dressing forceps. It is not always easy at this stage of anæsthesia to get into the mouth, as the lower-jaw muscles may not be relaxed. A proper tongue-forceps is not often at hand, and to tear the tongue-substance with sharp-toothed and yet slipping instruments, with the soreness and swelling that subsequently follow, is an abominable practice that should be abolished. The patient's chin and your own hands are always present, and it only needs knowledge of the method to apply it, and to secure prompt and speedy relief.

The form of inhaler used by Dr. Chisolm is a towel folded cone-form, with the apex of the cone open, so as to permit air to mingle freely with the chloroform vapor. During the administration the surgeon watches the patient's face, and, if the ears remain pink, the heart and lungs must be working properly; therefore there is no need for feeling the pulse. Any failure on the part of either of these organs can be seen in the change of countenance and complexion more quickly than it can be felt at the wrist. In Chisolm's clinic chloroform is denied to no surgical patient that needs a more efficient anæsthetic than a local one, such as cocaine. No pathological lesion in any part of the body deters him from the use of chloroform in an eye-operation, should it be required. He has operated about 1,500 times for cataract, and previous to the last two or three years, since cocaine came into use, he gave chloroform for all cataract operations. Probably the average age for cataract patients is sixty years; and most frequently they exhibit decided senile changes. Many of his cataract patients must have had fatty heart. So far, after thirty years of active surgical life, Chisolm says that in no single case has he had cause to regret that he chose chloroform as the anæsthetic. He always gives it in the presence of physicians—never alone.

His experience with four cases of sudden arrest of respiration, with failure of the heart's action, has made him a firm believer in the efficacy of inverted suspension for the restoration to life of patients apparently killed by chloroform; and he believes that many of the dead from chloroform might have been saved had the surgeon hung the patient up immediately by the feet, or held them in such a position that the head would have been very low, so as to get all the advantage of gravity in forcing the blood to the anæmic brain, instead of wasting time in applying hypodermic injections, cold-water splashings, spanking, electricity, etc., or even attempts at artificial respiration. Do any or all of these if you will, but hang the patient up first, and that immediately, as soon as the heart and lungs fail. It is the horizontal position that is fatal in chloroform-poisoning, and leads to death if the body is kept in it, as all reports of fatal cases with chloroform show. By suspended animation he refers to the complete arrest of all respiratory movements; not that very feeble state of heart and lung action, accompanied by pallor of the face, which only foreshadows approaching vomiting. This condition is common with many chloroform patients, but is only a signal that the basin must be held in readiness.

These views in regard to the value of inversion in suspended animation from anæsthetics, are in accordance with the results of Nélaton's experiments on chloroformed rats, which show that in chloroform narcosis, the respiratory and cardiac centres are weakened by an anæmic condition of the nervous apparatus, the exposed brains of animals bleaching as chloroform vapor was inhaled by them to complete anæsthesia. When a number of rats had been thoroughly narcotized with chloroform, those that were hung up by the tails slowly revived, while those left lying on the table died. And if, when animation began to show itself in the suspended animal, the rat was taken down too soon, breathing would again cease, and the rat would die unless immediately suspended again. When the animals were not already dead, suspension was the only means that would prevent death.

Of course no one that knows what he is about will ever give an anæsthetic without having some one present. Should there be any sudden and alarming weakening of the heart's action, and of respiration, for they always go together, hang up the patient without a minute's delay. Should the patient be bulky, and should there not be help enough present to elevate the foot of the table or bed, throw the head and body over the side of the table or bed, letting the head hang downwards to receive all the blood that can gravitate into it, holding on to the legs so that the body shall not slide to the floor. A still more efficient plan is, whether the patient is a man or a woman, while you stoop, throw their legs over your shoulders, hang on to their feet in front of you, and then lift yourself up. The patient's body, as you get upon your own feet, will hang from your back, with head down; and now, if you want more help you can call for it. But never leave the patient in a horizontal position while calling for help or doing anything else. If the patient vomit while inverted no harm can result, and the matters from the stomach cannot get into the larnyx.

